# The Acute Effect of Hemodialysis on Choroidal Thickness

**DOI:** 10.1155/2015/528681

**Published:** 2015-10-29

**Authors:** Osman Çelikay, Sinan Çalışkan, Tolga Biçer, Naciye Kabataş, Canan Gürdal

**Affiliations:** Ophthalmology Department, Dışkapi Yildirim Beyazit Training and Research Hospital, 06070 Ankara, Turkey

## Abstract

*Objective*. To determine the effect of hemodialysis (HD) on choroidal thickness (CT). *Methods*. The right eyes of 41 patients with end-stage renal disease (ESRD) undergoing HD were included. All patients underwent an ophthalmic examination, including CT measurement via optical coherence tomography, intraocular pressure (IOP), blood pressure, and body weight measurement immediately before and after a HD session. *Results*. Mean subfoveal choroidal thickness (SFCT) after HD decreased significantly from 254.59 ± 84.66 *µ*m to 229.34 ± 77.79 *µ*m (*p* < 0.001). CT at the temporal and nasal regions also decreased significantly after HD (both *p* < 0.001). IOP changes after HD were insignificant (*p* = 0.958). CT difference was insignificant in patients with diabetes mellitus (DM) and without DM before and after HD, respectively (*p* = 0.285 and *p* = 0.707). Stepwise multivariate linear regression analysis showed that diastolic blood pressure was the best fitted factor to explain the changes in CT (*r* = 0.327 and *p* = 0.040).  *Conclusion*. CT was decreased in the patients with ESRD following a HD session. This study suggested that the changes in CT may be related to the changes in systemic blood pressure.

## 1. Introduction

Hemodialysis (HD) sustains life for more than 1 million patients with end-stage renal disease (ESRD) worldwide and has been doing so since 1980 [1]. Therapeutic HD removes undesired solutes (potassium, urea, and phosphorus) primarily via diffusion and partially via convection through a semipermeable membrane. Membrane pore size determines the selective removal of solutes. Another goal of HD is maintaining the fluid balance by establishing a dry weight. Dry weight is defined as the lowest body weight that a patient can tolerate without the symptoms of hypotension until the next dialysis [2]. Dry weight is achieved by removing interdialytic weight gain via ultrafiltration during each HD session. The ultrafiltration rate and the degree of blood volume refill from the interstitial compartment during HD might cause intradialytic hypotension (IDH), which is defined as a relative or absolute decline in blood pressure (BP), as well as the presence of specific symptoms [3]. Patients undergoing HD cannot tolerate as great a decrease in blood volume as healthy people can. The presence of cardiac disease and autonomic neuropathy can exacerbate IDH and can compromise peripheral blood circulation [3].

It has been reported that the patients undergoing HD showed choroidal perfusion defect on fundus angiography (FFA) and indocyanine green angiography (ICGA) [4–6]. Traditional imaging modalities, such as ultrasonography (USG), FFA, and ICGA, are insufficient for quantitative assessment of the choroid [7]. More recent studies have focused on changes in choroidal thickness (CT) and retinal thickness following HD using different types of commercially available spectral domain optical coherence tomography (SD-OCT) in patients with ESRD. Although Jung et al. [8] reported a significant increase in CT after HD, two other studies reported a significant decrease in CT after HD [9, 10]. These inconsistent findings indicate that the effect of HD on CT is not clear. The present study, therefore, aimed at measuring changes in CT in ESRD patients after a HD session.

## 2. Materials and Methods

This prospective, cross-sectional study included 41 patients with stage 5 ESRD who were undergoing HD. Because of the high correlation between all parameters in the right and left eyes, only the patients' right eyes were analyzed. Pathologic conditions that could alter choroidal structure and CT, such as macular degeneration, smoking, ocular trauma, ocular surgery, ocular inflammation, and refractive errors outside −5 to +3 D, were excluded.

All patients were undergoing three 4-hour HD sessions each week. The bicarbonate used was diazole with a dialysate flow rate of 500 mL min^−1^ and blood flow rate of 250–300 mL min^−1^. Predialytic and postdialytic body weight were measured in each patient. Systolic and diastolic blood pressure (SBP and DBP, resp.) were measured every hour during HD. Ultrafiltration volume was noted following HD. To avoid HD induced hypoglycemia, all patients received 200 cc of 5% dextrose solution at the end of HD.

All patients underwent detailed ophthalmologic examination, including best-corrected visual acuity, intraocular pressure (IOP) before and after HD (Tonopachy NT-530P, Nidek Co., LTD., Tokyo, Japan), and lens status. CT scanning was performed using RTVue SD-OCT system (RTVue-XR 100 Avanti software v.6.1, Optovue, Inc., Fremont, CA, USA). The repeated OCT measurements were obtained by an operator (AÜ) at approximately 0700 and 1100, respectively, to avoid diurnal variations. The system utilized a wavelength of 840 ± 10 nm and was capable of 26,000 A-scans s^−1^. Tissue depth resolution was 5.0 *μ*m with transverse resolution of 15 microns. The device had a 40° Widefield Enface OCT reference scan.

Patients were seated at the front of the device to obtain reasonable choroidal images. The patients were informed to fixate the internal fixation light of the RTVue SD-OCT during image acquisitions. After the patients' heads were correctly positioned, a fovea-centered, 6 mm, horizontal high definition line scan (4096 A-scan/frame line) was obtained with the chorioretinal line mode. Postdialytic scans were obtained using the follow-up mode to obtain the images from the previous location. All images underwent manual segmentation by two researchers (Sinan Çalışkan and Tolga Biçer) who were blinded to the patients. RTVue manual measurement tools included calipers for delineating the boundaries of the choroid.

CT was measured perpendicularly from the outer edge of the retina pigment epithelium to the inner sclera, as previously reported [11]. CT was measured at the fovea and at 1.5 mm nasal, 3.0 mm nasal, 1.5 mm temporal, and 3.0 mm temporal to the fovea. Then, all parameters were measured by two researchers and averaged for analysis. The study was performed in accordance with the tenets of the Declaration of Helsinki; the study protocol was approved by the Dışkapi Yildirim Beyazit Training and Research Hospital Ethics Committee, Ankara, Turkey. Written informed consent was obtained from all patients.

### 2.1. Statistical Analysis

Data were analyzed using SPSS v.17.0 for Windows (SPSS, Inc., Chicago, IL, USA). The normality of the distribution of data was determined using the Kolmogorov-Smirnov test. All continuous data were normally distributed. There was a high correlation between the patients' right and left eyes; therefore, only right-eye data were used for further analysis. Descriptive statistics are presented as mean ± SD. For continuous data, Student's paired *t*-test and Pearson's coefficient test were utilized to compare pre-HD and post-HD data. The level of statistical significance was set at *p* < 0.05.

## 3. Results

The right eyes of 41 patients (61% female and 39% male) with ESRD were included. Mean age of the patients was 53.2 ± 14.6 years and mean duration of HD treatment was 42.81 ± 30.38 months. Eight of the 41 patients had diabetes mellitus (DM) (Table 1).

IOP changes after HD were insignificant (*p* = 0.958). Mean body weight decreased significantly from 65.77 ± 10.50 kg to 63.65 ± 10.41 kg after HD (*p* < 0.001). SBP and DBP decreased significantly following HD; the differences were 21.63 ± 23.06 mmHg and 14.00 ± 21.87 mmHg, respectively. IOP and systemic findings are shown in Table 2.

Mean subfoveal CT (SFCT) decreased significantly from 254.59 ± 84.66 *μ*m to 229.34 ± 77.79 *μ*m (*p* < 0.001) after HD. There was also a significant decrease in all measurement points from the fovea (Table 3).

There was no difference in CT between the patients with and without DM before and after HD. The mean SFCT was 241.88 ± 99.57 *μ*m and 257.67 ± 82.09 *μ*m, respectively, in the patients with DM and those without DM (*p* = 0.285). After HD, the mean SFCT was 203.0 ± 84.96 *μ*m and 235.73 ± 75.96 *μ*m, respectively, in patients with DM and those without DM (*p* = 0.707).

Associations between the difference in SFCT and the other parameters, including age, gender, DM, duration of HD, dry body weight, serum osmolarity, plasma osmotic colloid pressure, ultrafiltration volume, and the percentage of the change in IOP, SBP, DBP, and body weight, were evaluated (Table 4). There was a moderate positive correlation between changes in SFCT and the percentage of the changes in DBP (*r* = 0.311, *R* ^2^ = 0.097, and *p* = 0.047). Stepwise multivariate linear regression analysis showed that only change in DBP could explain 14% of the changes in SFCT (*R* ^2^ = 0.137; *p* = 0.022).

## 4. Discussion 

The primary goal of HD is to maintain the kidneys' excretory functions in ESRD patients. During a HD session, ultrafiltration removes excess fluid from plasma, which leads to blood volume depletion, an increase in the plasma protein concentration (increase in plasma colloid osmotic pressure), and a decrease in serum osmolarity. Blood volume depletion is equilibrated by vascular refilling from the interstitial and intracellular space [1, 12]. The choroid has a rich vascular network and the highest blood supply per organ area. In the present study, the changes in CT following a HD session were investigated. CT decreased significantly in the subfoveal, nasal, and temporal choroidal regions after HD.

Jung et al. [8] studied 28 eyes in 19 patients with ESRD and reported that SFCT increased following a HD session and was correlated with a decrease in SBP. They proposed that the increase in CT may be associated with the choroidal autoregulatory control of ocular hemodynamics, shifting of fluid, and molecules between the plasma and choroidal interstitium. Our results do not support this study. The difference between two studies may be related to the methodology used to measure CT and the demographic variation of the studies' population. Firstly, they used Image J software to measure SFCT. Chen and coauthors reported that Image J software showed better repeatability and agreement than the Heidelberg Eye explore software [13]. To the best of our knowledge, there was not any study which was comparing the intrinsic softwares of two OCT devices and Image J software. Secondly, coexisting systemic diseases such as DM may have the influence on the changes in CT after HD. But we did not find association between the presence of DM and CT changes. It may be related to the difference in the incidence of DM in two studies. While 46.4% of patients had DM in the first study, only 19.5% of the patients had DM in our study.

On the other hand, Yang et al. [10] observed a decrease in SFCT and IOP following HD. They suggested that choroidal blood flow is poorly autoregulated; a decrease in volume in the choroidal vascular bed may lead to a decrease in CT. Likewise, Ulaş et al. [9] reported a significant decrease in SFCT and nasal and temporal CT after HD but not in IOP levels. The researchers posited that the observed choroidal thinning may have been related to ultrafiltration-induced hypovolemia and increased plasma colloid osmotic pressure. Our findings regarding the CT changes were parallel to the results of both studies. The thinning of CT after HD may be caused by the changes in autoregulation of the choroidal blood flow and increased plasma colloid osmotic pressure. Additionally, in the present study, the CT thinning was correlated with a decrease in DBP. Furthermore, stepwise multivariate analysis showed that DBP is associated with 14% of the changes in SFCT.

Based on the association between the changes in CT and DBP, we hypothesized that the CT thinning may be a result of the increase in choroidal vascular and nonvascular smooth muscle contraction due to activation of the sympathetic autonomic nervous system. It is well known that retinal vascular circulation is autoregulatory and that choroidal circulation is controlled primarily by the extrinsic autonomic system [14]. The parasympathetic efferent nerves acting on vascular and nonvascular smooth muscle of the choroid tissue may lead to the increase in the choroidal blood flow. Sympathetic activation of choroidal smooth muscles leads to vasoconstriction and can cause CT to decrease [15]. It is well known that under physiological circumstances loss of blood volume activates the sympathetic system, initially leading to an increase in peripheral vascular resistance (due to constriction of resistance vessels), an increase in heart rate and myocardial contractility, and constriction of capacitance vessels [3]. During HD sympathetic activation it is necessary to initiate compensatory mechanisms and to maintain blood pressure, especially via an increase in the heart rate and peripheral vasoconstriction [16]. Although healthy individuals can tolerate a loss of 20% of blood volume before hypotension occurs, patients undergoing HD have a greater risk of hypotension and exhibit high interindividual variability and intraindividual variability in response to blood volume depletion. The main causes of hypotension as a complication of HD are thought to be acute hypovolemia during ultrafiltration and inadequate compensatory mechanisms, including autonomic dysfunction, diastolic and systolic dysfunction, decreased plasma osmolality, a decrease in extracellular fluid volume with inadequate plasma, impaired venous compliance, and decreased cardiac reserve [3, 16, 17]. Briefly, sympathetic system was activated to avoid hypotension in patients undergoing HD. This causes the constriction of choroidal blood vessels and nonvascular choroidal smooth muscle. Consequently, the choroidal vascular tonus changes lead to a decrease in CT.

In the present study, the changes observed in SFCT were positively correlated with changes in DBP. It is known that blood volume depletion causes diastolic dysfunction, whereas contractility dysfunction of the heart leads to systolic dysfunction. Barth et al. [18] reported that the mean DBP at the start of HD treatment and mean ultrafiltration volume were associated with intradialytic morbid events (mostly in hypotension-prone patients). We suggested that the decrease in DBP may be related to the IDH which activates sympathetic nervous system to avoid hypotension. DBP changes may be an indicator of the changes in SFCT.

Earlier findings on CT in DM patients have been inconsistent [19–21]. Recently, Farias et al. [22] reported significantly thinner CT in DM patients with microalbuminuria and suggested that decreased CT may be a sign of microvascular choroidal damage to the eye even before clinical signs become apparent. Yang et al. [10] observed that although CT was thinner in DM patients, the difference was insignificant in the patients with DM and without DM. Our findings support this study. We agree with earlier reports that suggest that a decrease in CT following HD may be associated with vascular damage and autonomic nervous system disruption in DM patients. The effect of HD on IOP remains unclear [23–25]. In the present study, IOP tended to decrease after HD, but the changes were insignificant. Plasma colloid osmotic pressure and serum osmolarity may have an effect on IOP changes, but additional research is needed to determine the precise effect of HD on IOP.

The present study has several limitations. Only predialysis serum osmolarity and plasma colloid serum osmotic pressure were measured, which limited our ability to analyze the correlation between changes in those parameters and SFCT. In a previous study, CT was shown to have a negative correlation with axial length [26]. In addition, axial length and anterior segment parameters such as anterior chamber depth that may have an influence on CT were not measured.

In conclusion, CT decreased after HD in patients with ESRD. We suggest that sympathetic activation triggered by blood volume depletion to prevent hypotension might cause choroidal vascular and nonvascular smooth muscle constriction, which leads to a decrease in CT. The decrease in DBP and SFCT following HD may be a consequence of systemic compensatory mechanisms. Additional large-scale randomized studies are needed to more fully determine the effect of HD on CT and the relationship between CT and changes in systemic parameters.

## Figures and Tables

**Table 1:** Demographic data.

	Mean + SD	Range
Age (years)	53.2 ± 14.6	28–82
Best corrected visual acuity (LogMAR)	0.14 ± 0.38	0–2
HbA1c (DM patients)	7.66 ± 1.20	6–9.20
Duration of HD (months)	42.81 ± 30.38	6–79
Dry weight (kg)	63.88 ± 10.48	43–83
Serum osmolarity (mOsm/L)	313.02 ± 9.48	296.74–330.52
Plasma colloid osmotic pressure (mmHg)	25.40 ± 2.25	16.43–29.49
Ultrafiltration volume (mL)	2647.22 ± 731.43	1350–4000

DM, diabetes mellitus; HbA1c, hemoglobin A1c; HD, hemodialysis.

**Table 2:** Effect of hemodialysis on the systemic hemodynamic parameters in ESRD patients.

	Before HD (mean + SD)	After HD (mean + SD)	Difference (mean + SD)	*p*
IOP (mmHg)	14.57 ± 3.06	14.55 ± 2.84	0.02 ± 2.39	0.958
Body weight (kg)	65.77 ± 10.50	63.65 ± 10.41	2.12 ± 0.97	<0.001
SBP (mmHg)	126.12 ± 19.40	104.49 ± 22.61	21.63 ± 23.06	<0.001
DBP (mmHg)	83.24 ± 23.26	69.24 ± 11.60	14.00 ± 21.87	<0.001

HD, hemodialysis; SD, standard deviation; IOP, intraocular pressure; SBP, systolic blood pressure; DBP, diastolic blood pressure.

**Table 3:** Effect of hemodialysis on the choroidal thickness in ESRD patients.

	Before HD (mean + SD)	After HD (mean + SD)	Difference (mean + SD)	95% CI of the difference		*p*
				Lower	Upper	
Nasal 3000 (*µ*m)	131.88 ± 61.86	115.61 ± 56.76	16.27 ± 19.37	10.1543	22.3823	<0.001
Nasal 1500 (*µ*m)	197.85 ± 73.92	183.32 ± 67.10	14.54 ± 17.01	9.1693	19.9039	<0.001
Foveal (*µ*m)	254.59 ± 84.66	229.34 ± 77.79	25.24 ± 28.98	16.0964	34.3914	<0.001
Temporal 1500 (*µ*m)	237.83 ± 83.97	214.90 ± 76.62	22.93 ± 23.17	15.6140	30.2396	<0.001
Temporal 3000 (*µ*m)	209.29 ± 64.17	194.90 ± 64.42	14.40 ± 25.27	6.0891	22.7004	0.001

HD, hemodialysis; SD, standard deviation; CI, confidence interval; *µ*m, micrometer.

**Table 4:** Multivariate linear regression analysis of subfoveal choroidal thickness changes after HD.

Factors	Subfoveal choroidal thickness change		
	Coefficients (95% CI)	*R* ^2^	*p* value
Age	0.27 (−0.189, 0.243)	0.002	0.801
Gender	1.100 (−5.282, 7.483)	0.003	0.729
DM	−6.169 (−13.780, 1.442)	0.064	0.109
HD duration	−0.010 (−0.117, 0.098)	0.001	0.853
Weight change	1.546 (−0.550, 3.641)	0.054	0.144
Dry body weight	−0.019 (−0.331, 0.293)	<0.001	0.903
Serum osmolarity	−0.196 (−0.543, 0.152)	0.035	0.262
Plasma osmotic colloid pressure	−0.851 (−2.314, 0.612)	0.037	0.246
Ultrafiltration volume	−0.001 (−0.006, 0.004)	0.005	0.694
SBP change	0.131 (−0.047, 0.309)	0.054	0.145
DBP change	0.147 (0.002, 0.293)	0.097	0.047
IOP change	0.180 (0.009, 0.351)	0.107	0.040

CI, confidence interval; DM, diabetes mellitus; HD, hemodialysis; SBP, systolic blood pressure; DBP, diastolic blood pressure; IOP, intraocular pressure.
